# Enhanced Antimicrobial Action of Chlorhexidine Loaded in Shellac Nanoparticles with Cationic Surface Functionality

**DOI:** 10.3390/pharmaceutics13091389

**Published:** 2021-09-02

**Authors:** Saba S. M. Al-Obaidy, Gillian M. Greenway, Vesselin N. Paunov

**Affiliations:** 1 Department of Chemistry and Biochemistry, University of Hull, Hull HU6 7RX, UK; sabasahib1976@gmail.com (S.S.M.A.-O.); GillianGreenway@gmail.com (G.M.G.); 2 Department of Chemistry, College of Science, University of Babylon, Hilla 51001, Iraq; 3 Department of Chemistry, Nazarbayev University, Kabanbay Batyr Ave. 53, Nursultan 010000, Kazakhstan

**Keywords:** antimicrobial nanocarriers, chlorhexidine, shellac, ODTAB, *E. coli*, yeast, microalgae, nanoparticles, Poloxamer 407

## Abstract

We report on an active nanocarrier for chlorhexidine (CHX) based on sterically stabilized shellac nanoparticles (NPs) with dual surface functionalization, which greatly enhances the antimicrobial action of CHX. The fabrication process for the CHX nanocarrier is based on pH-induced co-precipitation of CHX-DG from an aqueous solution of ammonium shellac and Poloxamer 407 (P407), which serves as a steric stabilizing agent. This is followed by further surface modification with octadecyl trimethyl ammonium bromide (ODTAB) through a solvent change to yield cationic surface functionality. In this study, we assessed the encapsulation efficiency and release kinetics of the novel nanocarrier for CHX. We further examined the antimicrobial effects of the CHX nanocarriers and their individual components in order to gain better insight into how they work, to improve their design and to explore the impacts of their dual functionalization. The antimicrobial actions of CHX loaded in shellac NPs were examined on three different proxy microorganisms: a Gram-negative bacterium (*E. coli*), a yeast (*S. cerevisiae*) and a microalgae (*C. reinhardtii*). The antimicrobial actions of free CHX and CHX-loaded shellac NPs were compared over the same CHX concentration range. We found that the non-coated shellac NPs loaded with CHX showed inferior action compared with free CHX due to their negative surface charge; however, the ODTAB-coated, CHX-loaded shellac NPs strongly amplified the antimicrobial action of the CHX for the tested microorganisms. The enhancement of the CHX antimicrobial action was thought to be due to the increased electrostatic adhesion between the cationic surface of the ODTAB-coated, CHX-loaded shellac NPs and the anionic surface of the cell walls of the microorganisms, ensuring direct delivery of CHX with a high concentration locally on the cell membrane. The novel CHX nanocarriers with enhanced antimicrobial action may potentially find applications in dentistry for the development of more efficient formulations against conditions such as gingivitis, periodontitis and other oral infections, as well as enabling formulations to have lower CHX concentrations.

## 1. Introduction

Chlorhexidine (CHX) is a synthetic antimicrobial agent that is widely used in antiplaque and antigingivitic formulations for treatment of periodontitis due to its wide-spectrum action against both Gram-negative and Gram-positive bacteria, as well as in other applications against yeasts, fungi and some viruses. Studies have revealed that CHX is able to neutralize pathogens such as Streptococcus aureus, *Porphyromans gingivalis* and *Prevotella intermedia* [1,2,3]. To minimize the potential side effects from using high concentrations of CHX derivatives, microcapsule formulations have recently been proposed for sustained CHX release based on poly ε-caprolactone (PCL), with 200–300 nm CHX-loaded PCL capsules showing similar minimal inhibitory concentration to *Staphylococcus epidermidis* as a chlorhexidine di-gluconate (CHX-DG) aqueous solution [4]. A single-emulsion-based solvent evaporation technique was used to fabricate microparticles with poly(dl-lactic-*co*-glycolic acid), chlorhexidine di-gluconate and a linking complex of either methylated-β-cyclodextrin or hydroxypropyl-β-cyclodextrin [5]. Seneviratne et al. [6] reported on mesoporous silica nanoparticles encapsulated with pure chlorhexidine and investigated their antimicrobial properties on planktonic bacteria, mono-species and mixed-species models of oral biofilms. Chlorhexidine hexametaphosphate nanoparticles (CHX-HMP NPs) measuring ~49 nm in diameter were used to coat dental implants. CHX-HMP NP-coated titanium surfaces displayed antimicrobial action against oral primary colonizing bacterium *Streptococcus gordonii* within 8 h. Recently, Al-Awady et al. [7,8] reported on the encapsulation of berberine and chlorhexidine into polyacrylic nanogel particles using a swelling–deswelling technique and showed that the functionalization of drug-loaded nanogel particles with a cationic polyelectrolyte improved their antimicrobial activity. The cationic coating controls the nanoparticles’ surface charges and promotes electrostatic adhesion to the microbial cell walls, which are negatively charged in aqueous media [9,10,11,12,13,14,15,16,17,18,19,20,21].

The effectiveness and side effects of the nanoformulations depend on the properties of the core materials used to fabricate and functionalize the nanocarriers. Shellac, which is the only pharmaceutical resin of insect origin, has a variety of applications as a moisture barrier material [22,23,24,25] in food products and cosmetics, and is widely used as an encapsulating pharmaceutical agent [26] and in enteric coatings for tablets [27]. Shellac has a complex composition of polar and non-polar polyhydroxy shelloic acids and is practically insoluble, even in slightly acidic aqueous media (pH < 7) [24,25,26,27,28]. Shellac’s biocompatibility makes it a very promising core material for the fabrication of nanoparticles for drug delivery applications [29,30,31,32]. Hamad et al. [24,25,26] reported on the use of colloid shellac microcapsules for cell encapsulation and pH-sensitive release. A colloidal shellac preparation was reported by Kraisit et al. [31], yielding 100–300 nm particles stabilized by chitosan, which was used for albumin encapsulation. Patel et al. [32] produced shellac colloids with particles measuring 150–300 nm using a solvent–anti-solvent technique, whereby the stabilization was achieved by using a xanthan gum. Recently, Al-Obaidy et al. [9,10] developed shellac NPs loaded with vancomycin and berberine, which were sterically stabilized using Poloxamer 407 (P407). P407 is a non-ionic triblock co-polymer consisting of poly(ethylene oxide)(PEO)-poly(propylene oxide)(PPO)-poly(ethylene oxide)(PEO), which is used as a solubilizing, emulsifying and dispersing agent in pharmaceutical formulations, in a similar manner to many other polymeric surfactants (pluronics) [33]. This nanoformulation showed far superior antimicrobial action compared to free vancomycin and berberine at the same concentrations. Weldrick et al. [34] took this approach a step further to encapsulate amphotericin B in protease-coated shellac nanoparticles, which were found to be effective in dispersing Candida albicans biofilms. A similar approach was found to work on bacterial biofilms [35]. Active antibiotic nanocarriers could become the key to breathing new life into old antibiotics and in overcoming antimicrobial resistance [11,12,36].

**Figure 1:**
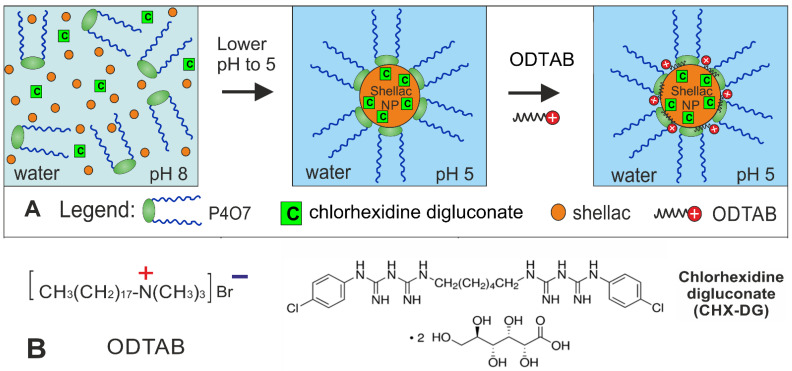
(**A**) Scheme of the process for preparation of steric stabilized shellac nanoparticles with a cationic surface functionality for targeted delivery of chlorhexidine digluconate (CHX-DG). The steric stabilization of the nanocarrier particles was achieved by using Poloxamer 407 (P407) at the co-precipitation stage with a pH drop from 8 to 5.5 followed by cationic surface functionalization by doping with the water-insoluble surfactant octadecyl trimethylammonium bromide (ODTAB). (**B**) Structural formulas of the ODTAB and CHX-DG as constituting materials for preparation of antimicrobial nanocarriers.

Here, we report on the fabrication of dual-functionalized, CHX-loaded, shellac-based nanocarriers with a high encapsulation efficiency and loading content of CHX and explore their efficacy against a range of proxy microorganisms. We compare the antimicrobial actions of the free CHX and CHX-loaded shellac NPs on *C. reinhardtii*, yeast and *E. coli* cells and examine the effects of each component included in the nanocarrier architecture in order to gain better insight into their role and function. We also explore the role of the P407 coating as a steric stabilizer, as well as its antimicrobial effect in the formulations [33]. Long polymer chains grafted (or physically adsorbed) on the nanoparticles’ surfaces can provide steric stabilization due to osmotic and elastic effects upon overlapping of polymer layers on their approaching surfaces in a good solvent [37]. Poloxamer 407 is an excellent steric stabilizer, as the hydrophobic polypropylene (PPO) chains are integrated with the shellac cores, leaving the hydrated polyethylene oxide (PEO) chains to form layers around the NPs. Here, the surface charge of these nanocarriers loaded with CHX was controlled by further functionalization using a water-insoluble cationic surfactant, which promoted electrostatic adhesion to the anionic surface of the microbial cell walls. Figure 1A shows the schematics of the preparation of the CHX-loaded shellac NPs. It gives the steps for the CHX encapsulation into the shellac nanoparticles and their subsequent surface functionalization. The loading of the CHX in the nanoparticle core is due to a combination of electrostatic and hydrophobic interactions between the CHX and the anionic shellac components (shelloic acids) in the nanocarrier. Figure 1B gives the structural formulas of the CHX-DG and cationic surfactant used for surface functionalization of the nanocarriers. The selection of the ODTAB for the subsequent surface functionalization allows direct deposition on the anionic shellac NPs’ surfaces, which are sterically stabilized by a layer of P407. This architecture of the nanocarrier proved to be successful, showing preserved stability despite the charge reversal. We also examined the release kinetics of CHX from the shellac nanocarrier and the enhancement of its antimicrobial action after functionalization with ODTAB. We demonstrated that this approach can strongly enhance the CHX antimicrobial action compared with a free CHX-DG. These steric-stabilized cationically functionalized nanocarriers allow the loaded CHX to be released directly into the cell membrane and boost its antimicrobial action by more than 10 times compared with the free CHX.

## 2. Materials and Methods

### 2.1. Materials

An aqueous solution of ammonium salt of shellac was used in this study, which was a gift from Stroever Schellackbremen (Bremen, Germany) and commercially sold as SSB Aqua Gold™ (25% solid). Chlorhexidine di-gluconate (20% solution in H_2_O) was obtained from Sigma-Aldrich, St. Louis, MO, USA. Poloxamer 407 (purified), chlorhexidine di-gluconate (20% in H_2_O) and fluorescein diacetate (FDA) were sourced from Sigma-Aldrich. Octadecyltrimethyl ammonium bromide (ODTAB, 97%) was supplied by Thermo Fisher Scientific, Waltham, MA, USA. Deionized water was produced using a Milli-Q water system (Merck Millipore, Burlington, MA, USA.) through ion exchange and reverse osmosis, the surface tension of which was 71.9 mNm^−1^ at 25 °C, with a measured resistivity of less than 18 MΩ cm^−1^. The BacTiter-Glo microbial cell viability assay was sourced from Promega, Madison, WI, USA.

*Escherichia coli* (Invitrogen MAX Efficiency™ DH10B™) was purchased from Thermo Fisher Scientific. The cells were grown as a suspension culture in a sterile Luria-Bertani medium (LB medium) [38] consisting of 0.5 g sodium chloride (99.8% Sigma-Aldrich), 0.5 g yeast extract and 1 g tryptone (from Thermo Fisher Scientific), dissolved in 100 mL autoclaved deionized water. *Saccharomyces cerevisiae* was sourced from Sigma-Aldrich. Here, 10 mg of lyophilized yeast cells was dispersed in 10 mL of deionized water. A 1 mL aliquot of the yeast suspension was inoculated in 100 mL of autoclaved YPD culture media consisting of 1.0 g of yeast extract, (Thermo Fisher Scientific), 2.0 g peptone (Sigma-Aldrich) and 2.0 g D-glucose (Thermo Fisher Scientific), then incubated at 30 °C for 24–48 h. *Chlamydomonas reinhardtii* (strain cc-124) was cultured in a TRIS–acetate–phosphate (TAP) medium made of TAP salts (phosphate buffer solution (PBS)) and Hunter’s trace elements solution (EDTA disodium salt, NH_4_Cl; (NH_4_)_6_Mo_7_O_24_·4H_2_O, MgSO_4_·7H_2_O, CaCl_2_·2H_2_O, ZnSO_4_·7H_2_O, H_3_BO_3_, CuSO_4_·5H_2_O, MnCl_2_·4H_2_O, CoCl_2_·6H_2_O, FeSO_4_·7H_2_O), all sourced from Sigma-Aldrich. The *C. reinhardtii* culture was grown at 30 °C and pH 7 in TAP media upon irradiation for 72 h with a white luminescent lamp with an intensity of 60 W m^−2^ with stirring using a magnetic stirrer. The produced *C. Reinhardtii* suspensions had a cell concentration of 4 × 10^5^ cells mL^−1^, as measured using a cell counter (Cellometer Auto X4, Nexcelom Bioscience, Lawrence, MA, USA). The *E. coli* stock suspension had a cell concentration of approximately 5 × 10^7^ cells mL^−1^.

### 2.2. Preparation of CHX-Loaded Shellac NPs with Dual-Surface Functionality

Shellac NPs were prepared by mixing 0.25 *w*/*v*% of the ammonium shellac and CHX-DG solution at pH 8 with different concentrations of CHX-DG and P407, followed by the reduction of the pH of the solution to 5 by dropwise addition of 0.01 M HCl under agitation with a magnetic stirrer. The concentration of the CHX-DG was varied in the P407 shellac starting solution with a constant ratio of 0.25 wt%/0.2 wt%. In order to enable adhesion between the CHX nanocarrier and the negatively charged microbial cell walls, the surface charge of the shellac NPs was reversed from negative to positive by subsequent surface doping with the cationic surfactant ODTAB. This was carried out by mixing 0.25 wt% ammonium shellac with 0.2% P407 and different concentrations of CHX-DG, after which the pH was lowered to 5 using 0.1 M HCl (aq.). Typically, 0.05–0.1 wt% CHX-DG was encapsulated in 0.25 wt% shellac NPs, which were then surface-functionalized with ODTAB to make the NPs cationic. The ODTAB, which is insoluble in water, was added dropwise to the shellac NP suspension as a 3 wt% solution in ethanol. The size and the zeta potential of the CHX-loaded shellac nanoparticles were measured using a Zetasizer Nano ZS (Malvern Panalytical Ltd., Malvern, UK).

### 2.3. CHX Encapsulation Efficiency, Drug Loading Content and Release Kinetics Measurements

UV-vis (FLUOstar Omega spectrophotometer, BMP Labtech Ltd., Aylesbury, UK) and FTIR spectroscopy (Nicolet 360 FT-IR, Thermo Scientific, Hemel Hempstead, UK)) were used to explore the encapsulation of CHX within the novel shellac NPs after removing the excess of CHX-DG using a centrifugation–washing process, which was carried out three times. The encapsulation efficiency and the percentage of the CHX loaded into the NPs were determined indirectly by measuring the unencapsulated amount of chlorhexidine using the linear regression equation calculated from the calibration curve of CHX (see Figure S1, ESI). In vitro release studies were conducted to monitor the concentration of CHX released from the shellac NPs at a fixed pH. The suspension of CHX-loaded shellac NPs was placed into a dialysis bag with a pore size of 2.5 nm, which allowed the CHX to be released from the nanoparticles and diffuse through its pores. The dialysis bag was placed into a beaker with either a phosphate buffer solution of pH 5.5 or a phosphate saline buffer (PBS) solution at pH 7.4. After this, the concentration of CHX released was measured spectrophotometrically. All release experiments were carried out in triplicate. The cumulative percentage of CHX release was calculated using the following equation:

$$\%\;\mathrm{in}\;\mathrm{vitro}\;\mathrm{CHX}\;\mathrm{released}=\frac{M_{released}}{M_{total}}\times 100$$

where $M_{released}$ is the amount of CHX released from the shellac NPs at time *t* and $M_{total}$ is the whole amount of the CHX loaded into shellac NPs.

### 2.4. Testing the Antimicrobial Action of CHX Formulations on E. coli

We cultured *E. coli* in LB culture media. An aliquot of 50 mL of the bacterial cell culture was centrifuged for 5 min at 3000 rpm and the cell pellet was redispersed in 50 mL of deionized water. CHX formulations of different concentrations were prepared in 5 mL aliquots and added to 5 mL of the *E. coli* culture, then gently shaken to mix. The mixtures were incubated for 15 min, 2 h and 4 h, respectively, under the same conditions as the control (non-treated E. coli culture). After the treatment the *E. coli* sample was centrifuged to remove the excess of CHX, then the cell pellet was resuspended in 1 mL of deionized water. Next, a 100 μL aliquot of each sample was then dispensed into a 96-well plate (opaque) and mixed with 100 μL Promega Cell Titer-Glo luminescent cell viability assay reagent, equilibrated at 25 °C for half an hour, then the luminance was measured using a Thermo Scientific Fluroskan Ascent FL.

### 2.5. Testing the Antialgal Action of CHX Formulations on C. reinhardtii

*C. reinhardtii* was cultured in TAP media with a typical cell count of 5 × 10^5^ cells mL^−1^. Here, 50 mL of culture was separated to test the cell viability by centrifugation and the cells were pelletized and resuspended in 50 mL of deionized water. A 0.5% *w*/*v* fluorescein diacetate (FDA) solution in acetone was prepared and 20 μL of this solution was mixed with 1 mL of the cell sample, then the mixture was vortexed at 1500 rpm for 10 min at room temperature in dark conditions to avoid photobleaching. This was also carried out for the algal cells incubated at room temperature for 2 h and 4 h, respectively. The cell viability was determined using a Nexellom Auto X4 cell counter (Nexcelom Bioscience, Lawrence, MA, USA). Next, 20 μL of the sample was loaded into a cellometer counting chamber and the numbers of fluorescent (viable) and non-fluorescent (non-viable) cells were measured using bright field and fluorescent microscopy, respectively. These measurements were carried out in triplicate.

### 2.6. Testing the Antimicrobial Action of CHX Formulations on S. cerevisiae

*S. cerevisiae* was cultured with an initial concentration of 0.01 g of lyophilized *S. cerevisiae* per 100 mL of YPD media. For cell viability measurements, a 50 mL aliquot was removed from the culture media by centrifugation, then pelletized and redispersed in deionized water. Next, 1 mL of washed *S. cerevisiae* sample was pipetted into a tube, 20 μL of the 0.5 % *w*/*v* FDA stock solution in acetone was added and the mixture was vortexed for 30 min at 1500 rpm. The same procedure was used for the cells exposed to the treatments at room temperature for 2 h and 4 h. The cell viability was measured using the Nexellom Auto X4 cell counter as described above.

## 3. Results and Discussion

### 3.1. CHX Encapsulation and Characterization of CHX Loaded within Shellac NPs

**Figure 2:**
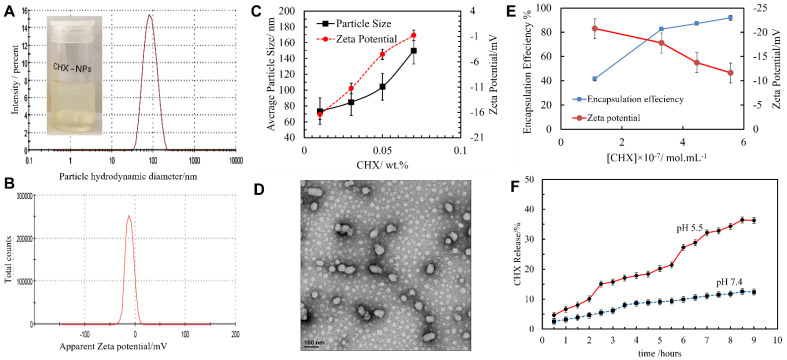
(**A**) The hydrodynamic diameter distribution of the shellac NPs. (**B**) The zeta potential distribution of the shellac NPs for a composition of 0.25:0.2 wt% of shellac/P407 obtained by co-precipitation by dropping the pH from 8 to pH 5 in deionized water. (**C**) The average diameter and zeta potential of the shellac NPs as a function of the pH of the aqueous media. The particles were not coated with ODTAB in this experiment. (**D**) A TEM image of the non-loaded shellac NPs for a solution consisting of 0.25 wt% shellac with 0.2 wt% P407. (**E**) The encapsulation efficiency levels (as percentages) of different concentrations of CHX-loaded shellac nanoparticles at pH 5 (*n* = 3). (**F**) The percentages of in vitro CHX release as a function of time for two different values of pH (5.5 and 7.4). The measurements were carried out using a Perkin–Elmer UV-visible spectrophotometer at wavelengths in the range of 200–700 nm (*n* = 3).

CHX-DG was encapsulated within shellac NPs by mixing the three components shellac, P407 and chlorhexidine at pH 8, then decreasing the pH to 5 to protonate the shellac carboxylic groups and to decrease their solubility so that they precipitated as nanoparticles. P407 is a steric stabilizer for the formed colloid that limits the particle growth. Figure 2A,B show typical the particle size and zeta potential distributions of the shellac NPs with the composition mentioned in the previous section. The average hydrodynamic diameter of the CHX-loaded shellac NPs was 79 ± 30 nm, with an average zeta potential of −11 ± 8 mV before coating with ODTAB. Note that the average size of the shellac NPs increased from 66 to 79 nm, which indicated that there was an interaction between the shellac components in the NPs and the embedded chlorhexidine. Simultaneously, the zeta potential decreased from −18 mV to −11 mV due to the interaction between the shelloic acid’s carboxylic groups and the CHX cationic centers (see Figure 2C). It is not expected that the size of the produced shellac nanoparticles would depend on the length of the hydrophobic hydrocarbon tail of the cationic surfactant, since the shellac particles are already formed and covered by a dense layer of Poloxamer 407 before the cationic surfactant is introduced as a dopant. Our results show that the ODTAB mainly affects the surface charge of the shellac NPs and causes their charge reversal. The data presented in Figure 2E show that at high concentrations of CHX, the size of the shellac NPs started increasing due to the increased amounts of loaded CHX, reaching approximately 150 nm at 0.05 wt% CHX, while the zeta potential of the NPs decreases from −20 mV to less than −1 mV, resulting from the increasing amount of the CHX cations containing 10 N atoms with two cationic centers. The decrease in the zeta potential could also be indicating that the CHX has intercalated within the shellac NPs and altered their surfaces. A transmission electron microscopy (TEM) image of non-loaded shellac NPs is shown in Figure 2D. TEM images of CHX-loaded and non-loaded shellac NPs are presented in Figure S7 (ESI). The image indicates that the CHX-loaded shellac NPs have roughly a spherical shape.

### 3.2. FTIR and UV-Vis Spectra of Shellac NPs, Free CHX and CHX-Loaded Shellac NPs

Fourier transform infrared (FTIR) spectroscopy was used to examine the intercalation of CHX into the shellac NPs. The FTIR spectrum of shellac NPs (Figure S2A, ESI, brown line) was compared with the CHX spectrum (Figure S2A, blue line), where the principal stretching vibrations from 3300 cm^−1^ to 3500 cm^−1^ are for the N–H group and the stretching bands at 2850 cm^−1^ to 3000 cm^−1^ belong to the aliphatic C–H group. The peak linked to the stretching vibration band for the aliphatic C=N group is at 1672 cm^−1^. The peaks assigned to the C=C group in the aromatic ring are at wavelengths ranging from 1450 cm^−1^ to 1550 cm^−1^ and at 1251 cm^−1^, which is linked to the stretching vibration of the aliphatic amine (C–N) group. These spectra agree with the results of other studies in the literature [39,40,41]. The spectrum of the CHX-loaded shellac NPs (Figure S2A, ESI, green line) shows a broad peak belonging to the overlapping of O–H and N–H bands for both shellac components and the CHX. The CHX bands overlapped with the shellac NP bands and with a small offset, ascribed to the C=O, O–H and C–O stretching at 1710, 1342 and 1240 cm^−1^, respectively. The UV-vis spectra of shellac NPs, CHX-DG and CHX-loaded shellac NPs are given in Figure S2B (ESI). The spectrum of the shellac NPs (the red line) shows peaks at 300 to 200 nm due to the different shellac components, with no distinct maximum wavelength, while the CHX spectrum (the green line) shows three absorption peaks, all in the UV region at wavelengths of 255, 231 and 209 nm, respectively. The P407 coating did not show any absorption peaks above 200 nm. The black line represents the absorbance spectrum of the CHX-loaded shellac NPs, with two peaks appearing at 260 and 227 nm, respectively, belonging to both the absorbance of CHX and shellac. This indicates that there is an interaction between the CHX and the shellac components in the CHX-loaded shellac NPs.

### 3.3. CHX Encapsulation Efficiency and CHX Release Studies

Figure 2E shows that the CHX encapsulation efficiency in the shellac NPs reached 92% of the total CHX concentration, with a loading percentage of 16%. Table S1 (ESI) shows the loading content of the shellac NPs at several different CHX-DG concentrations. The high encapsulation efficiency is due to the strong electrostatic interaction between the shelloic acids and the CHX cations. Figure 2F shows that the cumulative percentage of released CHX at pH 5.5 is higher than at pH 7.4, which is explained as follows. At pH 5.5 the carboxylic groups of the shellac nanoparticles are better protonated, which increases the release of CHX, reaching ~36% after 8 h, while at pH 7.4 the shelloic acid groups are more deprotonated and interact more strongly with the CHX cations, so that the release of the CHX is slower amounting, to only 12% after 8 h.

### 3.4. Antimicrobial Activity of Free CHX and CHX-Loaded Shellac NPs on E. coli

**Figure 3:**
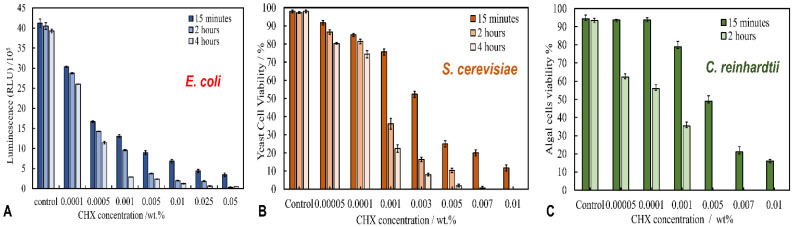
(**A**) The relative luminescence unit, which represents the viability of *E. coli* cells incubated at pH 5.5 with different concentrations of an aqueous solution of free chlorhexidine at different incubation times of 15 min, 2 h and 4 h at room temperature using bactiter luciferase reagent (*n* = 3). (**B**) The viability of yeast cells upon incubation at pH 5.5 with varying concentrations of free chlorhexidine at room temperature with 15 min, 2 h and 4 h incubation time using FDA assay (*n* = 3). (**C**) The viability of algal cells (*C. Reinhardtii*) upon incubation with varying concentrations of free chlorhexidine at room temperature for 15 min and 2 h incubation time at pH 5.5 using FDA assay (*n* = 3).

To study the antibacterial activity of free CHX and CHX-loaded shellac NPs, various concentration equivalents were incubated with a sample of *E. coli*. The *E. coli* cells were treated with BacTiter-Glo^®^ Luciferase reagent, then washed so that their viability could be evaluated in terms of luminescence. Figure 3A shows the effects of free CHX-DG, which exhibits a strong antibacterial effect at very low concentrations after 15 min of treatment. The cell viability was estimated from the relative luminescence units (RLU), which are proportional to the residual ATP content of the cells and indicate the level of their metabolic activity. The RLU sharply decreased to 9 × 10^5^, 7 × 10^5^, 4 × 10^5^ and 3 × 10^5^ RLU at CHX-DG concentrations of 0.005, 0.01, 0.025 and 0.05 wt%, respectively, relative to the viability of the control sample, which had 4.1 × 10^6^ RLU. The *E. coli* viability declined between 2 h and 4 h of treatment and approached zero at CHX-DG concentrations above 0.01 wt%. Note that the antibacterial effect of CHX decreased upon its encapsulation within shellac NPs without the cationic ODTAB coating. Figure S3A (ESI) shows that the bacterial cell viability only slightly decreased after 6 h of treatment. With 0.01 wt% CHX loaded in the shellac NPs, the *E. coli* viability declined from 40 × 10^5^ RLU for the control sample (no treatment) to 2.7 × 10^6^, 1.85 × 10^6^, 1.65 × 10^6^ and 1.25 × 10^6^ RLU after 15 min, 2 h, 4 h and 6 h, respectively. The decrease of the antibacterial effect of CHX after loading it within shellac NPs is explained by the negatively surface charge of the shellac NPs, which are repelled by the negatively charged bacterial cell walls, as well as the slower release of CHX from the NPs due to the strong interactions between shellac carboxylic acid groups and the CHX cationic centers. The SEM images reflect these findings, as shown in Figure S4 (ESI). In Figure S4B (ESI), the treatment with free CHX-DG caused some damage to the *E. coli* cell wall in comparison to the control shown in Figure S3A (ESI), although the shellac NPs with the encapsulated CHX seem to be repelled by the negatively charged bacterial cell wall and do not accumulate there (see Figure S4C,D). To enhance the accumulation of nanocarriers on the bacterial cell wall, the shellac NPs were further functionalized to change the surface charge from negative to positive by coating them with a cationic surfactant.

### 3.5. Anti-Yeast Activity of Free CHX and CHX-Loaded Shellac NPs on S. Cerevisiae

The antifungal activity of free chlorhexidine- and chlorhexidine-loaded shellac NPs was investigated on yeast (*S. cerevisiae*) cells. Figure 3B presents the viability of *S. cerevisiae* cells after treatment with aqueous solutions containing different concentrations of free CHX-DG. Figure 3B shows that after 15 min of incubation, the yeast cell viability decreased by half at 0.003 wt% and by quarter at 0.005 wt% CHX in comparison with the control, which was 98%. Most yeast cells in the sample were killed after 2 h of treatment at a concentration of free CHX above 0.005 wt%. After 4 h of treatment with 0.001 and 0.003 wt% free CHX-DG, the yeast cell viability declined to 22% and 8%, respectively; however, by encapsulating CHX within shellac NPs, the antimicrobial activity was greatly reduced. Figure S3B shows that the yeast cell viability gradually decreased after 6 h of treatment from 98% for the control sample to 75%, 70% and 65% at 0.005, 0.007 and 0.01 wt% overall concentrations of CHX encapsulated in shellac NPs, respectively. These data indicated that the anti-yeast action of CHX was decreased by about 75% after encapsulation within shellac NPs. This was a similar result as that obtained for *E. coli,* with a similar explanation regarding the anionic character of the shellac NPs; however, in this case the lack of effect was exacerbated by the fact that yeast cells have a thick cell wall around their cell membrane, which impairs the action of the CHX, in addition to the negative surface charge of the NPs, which leads to a significant repulsion between the cell membrane and NPs. Along with these effects, the CHX release is slow (about 36% of total amount of CHX was released after 9 h at pH 5.5) due to the strong interaction between the deprotonated shelloic acid carboxylic groups and the CHX cations in the NPs. These results were also supported by the SEM images (see Figure S5C,D, ESI), which indicate that the damage inflicted by the free CHX-DG treatment has a much greater effect on yeast cell membrane than the shellac NP-encapsulated CHX (Figure S5E,F, ESI) in comparison with the non-treated yeast control samples (Figure S5A,B, ESI).

### 3.6. Antialgal Activity of Free CHX and CHX-Loaded Shellac NPs on C. reinhardtii

Figure 3C and Figure S3C show the antialgal actions of free CHX and CHX-loaded shellac NPs on the *C. reinhardtii* microalgae for different treatment times at 25 °C. Note that the free CHX exhibits high toxicity for microalgae cells, as their cells viability was reduced after 15 min of treatment. This was most evident with 0.01 wt% of free CHX, as the viability went from 94% for the control sample down to 16%. After 2 h of treatment, the microalgae viability sharply declined and practically all microalgae cells died at CHX concentrations above 0.005 wt%. In contrast, the antialgal activity of CHX decreased slightly after its encapsulation within shellac NPs, as seen in Figure S3C. This reduction in the antialgal activity of CHX is due to the repulsion between the anionic surface of the CHH-loaded shellac NPs and the anionic cell membrane, which does not allow the loaded CHX to be released in the vicinity of the cell wall, combined with its slow release. As explained previously, this is attributed to the strong interaction between the shelloic acids and the CHX cationic nitrogen atoms. The SEM images of the treated microalgae cells presented in Figure S6 (ESI) indicate that the free CHX-DG treated cells shrank and appeared to be wrinkled (Figure S6C–F, ESI) when compared with the control sample of non-treated cells, as shown in Figure S6A,B (ESI). Figure S6E,F (ESI) shows the microalgal cells after incubation with 0.005 wt% of CHX-loaded in shellac NPs; as would be expected, there was a less pronounced effect than the treatment with equivalent concentration of free CHX-DG.

### 3.7. Antibacterial Activity of ODTAB-Coated, CHX-Loaded Shellac NPs on E. coli

**Figure 4:**
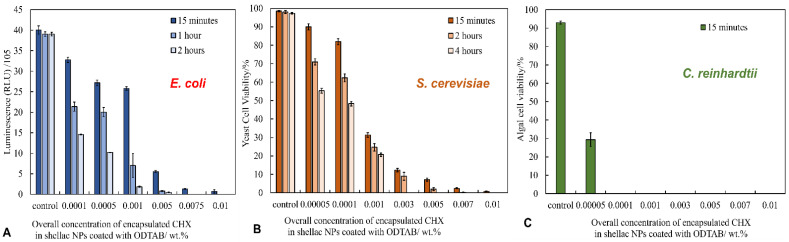
(**A**) The levels of antibacterial activity against *E. coli* cells of different concentrations of CHX-loaded shellac NPs coated with ODTAB at 15 min, 1 h, and 2 h. The cell viability is represented by relative luminescence units. These solutions were prepared from 0.03 wt% CHX-loaded shellac NPs coated with 0.05 wt% ODTAB (*n* = 3). (**B**) The cytotoxic effects of different concentrations of CHX-loaded shellac NPs coated with ODTAB upon incubation with yeast cells at room temperature at 15 min, 2 h, and 4 h using FDA assay. The suspensions were prepared from 0.05 wt% stock solution of CHX-loaded shellac NPs coated with 0.05 wt% ODTAB (*n* = 3). (**C**) The viability of algal cells (*C. Reinhardtii*) upon incubation at pH 5.5 with different amounts of CHX-loaded shellac NPs coated with ODTAB measured by using FDA assay after washing the cells from the treatment at room temperature at 15 min of incubation time. The solutions were prepared from stock solution of 0.05 wt% CHX-loaded shellac NPs coated with 0.05 wt% ODTAB (*n* = 3).

**Figure 5:**
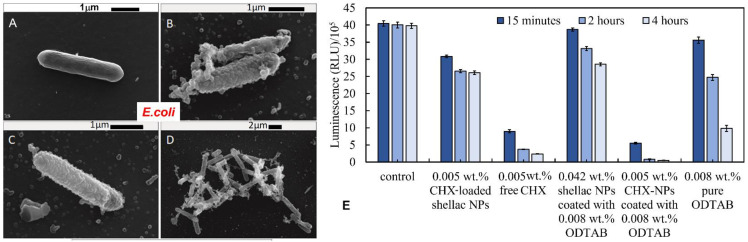
SEM images of *E. coli* cells. (**A**) Control sample of *E. coli* cells. (**B**–**D**) *E. coli* cells incubated with 0.005 wt% chlorhexidine-loaded shellac NPs coated with ODTAB at pH 5.5 at room temperature. (**E**) The antimicrobial activities of 0.005 wt% CHX-loaded 0.042% shellac NPs coated with 0.008 wt% ODTAB towards *E. coli* cells compared with the antimicrobial activity of free CHX and shellac NP-encapsulated CHX without ODTAB coating, 0.008 wt% ODTAB-coated 0.042 wt% shellac NPs without CHX payload, as well as free ODTAB at the same concentration (0.008 wt%). The incubation was also achieved through incubating each concentration with a fixed amount of *E. coli* cells at pH 5.5 at room temperature using BacTiter Glo™ Luciferase assay (*n* = 3).

We then examined the antibacterial activity of CHX-loaded shellac NPs after coating with the cationic surfactant ODTAB. The coating was carried out to reverse their surface charge from negative to positive, which was expected to enhance the electrostatic adhesion between the NPs and the cell wall. We performed the experiments on *E. coli* cells at pH 5.5. Figure 4A shows that after only 15 min of treatment, the CHX loaded in shellac NPs coated with ODTAB did not affect significantly the *E. coli* cells with a concentration range 0.0001–0.001 wt% CHX, while at higher CHX concentrations, the cell viability decreased significantly. After 2 h of treatment, most *E. coli* cells died above the concentration of 0.001 wt% of CHX-loaded in shellac NPs coated with ODTAB. The *E. coli* cell viability decreased strongly after 4 h of treatment, giving a value of 3.9 × 10^6^ RLU compared to the control sample value of 5 × 105 RLU. All cells lost their viability above 0.005 wt% of CHX loaded in shellac NPs coated with 0.008 wt% ODTAB. Figure 5B–D shows SEM images of *E. coli* cells treated with 0.005 wt% CHX-loaded shellac NPs coated with ODTAB for 2 h. Figure 5A shows an SEM image of non-treated *E. coli* as a control. Note that the CHX-loaded shellac NPs coated with ODTAB strongly adhere to the bacterial cells compared with the uncoated ones (see Figure S4C,D, ESI). Figure 5E shows a comparison of the effects of the free CHX-DG, uncoated CHX-loaded shellac NPs and ODTAB-coated, CHX-loaded shellac NPs as treatments for the *E. coli* cells. We also compared the antimicrobial activity of ODTAB-coated shellac NPs (no CHX) and free ODTAB at the same overall concentration of ODTAB as in the shellac NP formulations. The uncoated CHX-loaded shellac NPs showed weaker antibacterial effects than the free CHX-DG after 4 h of treatment, whilst as expected, the shellac NPs coated with ODTAB had a profound effect on the cell viability due to their cationic surface. A considerable increase in the antibacterial effect was noted after treating *E. coli* cells with ODTAB-coated 0.005 wt% CHX loaded in shellac NPs at the same treatment duration.

### 3.8. Anti-Yeast Activity of ODTAB-Coated, CHX-Loaded Shellac NPs on S. cerevisiae

**Figure 6:**
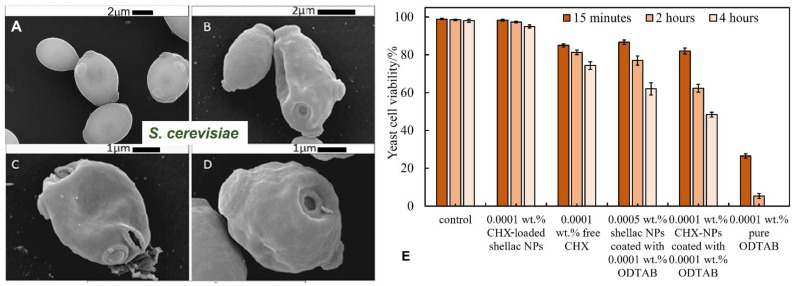
SEM images of yeast cells. (**A**) A control sample of the yeast cells. (**B**–**D**) Yeast cells incubated with 0.005 wt% CHX-loaded shellac NPs coated with 0.005 wt% ODTAB after 2 h at room temperature. (**E**) The viability of yeast cells upon incubation with 0.0001 wt% CHX-loaded shellac NPs, 0.0001 wt% free CHX, 0.0005 wt% shellac NPs coated with 0.0001 wt% ODTAB, 0.0001 wt% CHX NPs coated with 0.0001 wt% ODTAB and 0.0001 wt% pure ODTAB at pH 5.5 and at room temperature (*n* = 3).

Figure 4B shows the effect of ODTAB-coated, CHX-loaded shellac NPs on *S. cerevisiae* after up to 4 h of treatment. One can see a pronounced effect on the yeast cell viability after 15 min of exposure at 0.003, 0.005, 0.007, and 0.01 wt% of CHX loaded in shellac NPs coated with ODTAB. The cell viability was sharply reduced from 98.5% for the control sample to 12%, 7%, 2.4% and 0.8%, respectively, for the aforementioned concentrations of CHX. After 4 h of exposure, the cell viability strongly decreased above 0.005 wt% of CHX loaded in shellac NPs coated with ODTAB. The SEM images shown in Figure 6B–D demonstrate that the cell walls were severely impacted after 2 h of exposure to 0.005 wt% CHX loaded in shellac NPs coated with 0.005 wt% ODTAB (see Figure 6A shows control with no treatment). Figure 6E gives a comparison of the effects of 0.0001 wt% free CHX-DG and 0.0001 wt% CHX loaded in shellac NPs coated with 0.0001 wt% ODTAB on the yeast cells. We compared these results with the antimicrobial activity of 0.0001 wt% CHX encapsulated in shellac without coating, 0.0001 wt% ODTAB-coated 0.0005 wt% shellac NPs without any CHX load and 0.0001 wt% free ODTAB. The results showed that after coating of the CHX-loaded shellac NPs with ODTAB, the anti-yeast effect of the CHX increased due to the strong attraction between cationic CHX-loaded shellac NPs coated with ODTAB and the cell walls, amplifying the amount of locally delivered CHX ions directly on the yeast cells’ surfaces.

### 3.9. Antialgal Activity of ODTAB-Coated, CHX-Loaded Shellac NPs on C. reinhardtii

**Figure 7:**
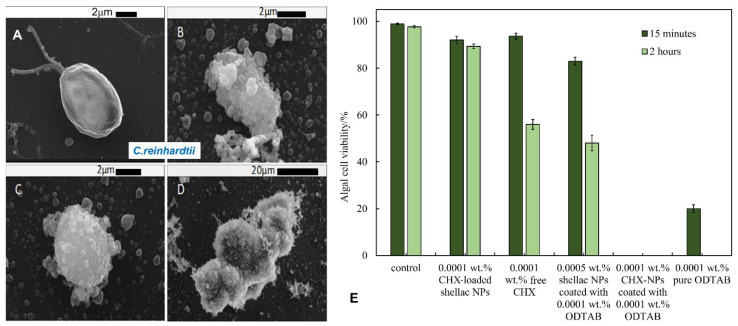
SEM images of *C. reinhardtii* cells. (**A**) A control sample of microalgae cells. (**B**–**D**) *C. reinhardtii* microalgae cells incubated with 0.005 wt% CHX-loaded shellac NPs coated with 0.005 wt% ODTAB after 2 h of incubation time at room temperature. (**E**) The *C. reinhardtii* algal cells viability upon incubation with 0.0001 wt% CHX-loaded shellac NPs, 0.0001 wt% free CHX, 0.0005 wt% shellac NPs coated with 0.0001 wt% ODTAB, 0.0001 wt% CHX-NPs coated with 0.0001 wt% ODTAB and 0.0001 wt% pure ODTAB at pH 5.5 and room temperature (*n* = 3).

The antialgal activity of ODTAB-coated, CHX-loaded shellac NPs was studied on *C. reinhardtii* cells at different equivalent CHX concentrations and treatment durations. Figure 4C represents the microalgal cell viability after 15 min treatment with various concentrations of CHX loaded in shellac NPs coated with ODTAB. One can see that all of the cells lost their viability after 15 min of treatment with formulations containing the above 0.0001 wt% CHX loaded in shellac NPs coated with ODTAB. Figure 7B–D shows SEM images of the microalgal cells after being incubated with 0.005 wt% CHX loaded in shellac NPs coated with 0.005 wt% ODTAB for 2 h compared with the control (Figure 7A). The images in Figure 7C,D indicate that the cells were clustered, while the cationic NPs accumulated on the cell walls due to the positively charged surfaces of the NPs loaded with CHX coated with ODTAB. Figure 7E shows the comparison of the antialgal activities of 0.0001 wt% CHX loaded in shellac NPs, 0.0001 wt% free CHX, 0.0005 wt% CHX loaded in shellac NPs coated with 0.0001 wt% ODTAB, 0.0001 wt% CHX-loaded shellac NPs coated with 0.0001 wt% ODTAB and 0.0001 wt% free ODTAB. The data indicate that there was a drop in the antialgal effect of CHX when loaded within shellac NPs in comparison with effect of the free CHX, while there was a profound change in the antialgal action of the encapsulated CHX after coating the nanocarrier with ODTAB. Note that most other CHX nanocarriers reported in the literature had negative surface charges [42,43,44,45]. The cationic surface of the ODTAB-coated NPs attracts them to the negatively charged algal cell membrane and subsequently delivers higher loads of CHX ions on their membranes.

## 4. Conclusions

In this study, we explored the antimicrobial effects of CHX encapsulated in dual-functionalized shellac NPs on three proxy microorganisms, including bacteria (*E. coli*), microalgae (*C. reinhardtii*) and yeast (*S. cerevisiae*). The NPs were sterically stabilized by a Poloxamer 407 coating produced during the formulation stage of the nanocarrier. The results showed that without a cationic coating, the CHX-loaded shellac NPs did not express pronounced effects on these microorganisms, apart from a minor effect on the microalgae. Free CHX showed significant antimicrobial effects on E. coli, yeast and microalgae, although the antmicrobial action was suppressed when CHX was encapsulated within shellac NPs owing to the high interaction between shelloic acid carboylic groups and the CHX ions, as well as the electrostatic repulsion between the anionic shellac NPs’ surfaces and the microbial cell membrane. To promote NP–cell adhesion, the NPs were further coated with the cationic surfactant ODTAB to generate a cationic surface for the shellac nanocarriers. After coating the CHX-loaded shellac NPs with ODTAB, their antimicrobial effect received a boost due to their accumulation on the microbial cell wall, as indicated by SEM images. This allowed CHX to be released directly in the vicinity of the cell membrane and to kill the cells even at a very low overall CHX concentration. The ODTAB-coated, CHX-loaded shellac NPs displayed significantly higher antimicrobial effects on *C. reinhardtii* than on *S. cerevisiae* and *E. coli,* which was linked to the thickness and architecture of their cell walls. When CHX was loaded with shellac NPs and coated with ODTAB, its antimicrobial effects were amplified by more than 10 times compared with other nanocarriers. This was attributed to the increased attraction of the nanocarriers to the bacterial cell membrane, allowing even very low overall concentrations of CHX to kill the bacterial cells. The designed shellac nanocarriers were sterically stabilized with P407, loaded with CHX and coated with ODTAB. They showed significant antimicrobial activity, which was higher than or on par with CHX formulations reported in the literature. This nanotechnology has the potential to produce more effective antiseptic agents, better dentistry formulations for control of plaque formation, better wound dressings, as well as innovative antialgal and antibiofouling formulas.

## Supplementary Materials

The following are available online at https://www.mdpi.com/article/10.3390/pharmaceutics13091389/s1. Figure S1: The UV-vis calibration curve at 255 nm of different concentrations of chlorhexidine di-gluconate (CHX-DG) in deionized water, (*n* = 3), Figure S2: (A) The Fourier Transform-IR spectrum of chlorhexidine, 0.03 wt%. CHX-loaded shellac NPs, and shellac NPs. (B) The absorption spectrum of chlorhexidine, free shellac, chlorhexidine loaded shellac NPs and Poloxamer 407 using UV-Vis spectrophotometry, Figure S3: (A) The relative luminescence unit representing *E.coli* cells viability incubated at pH 5.5 with different overall concentrations of CHX-loaded shellac NPs at different incubation time 15 min, 2 h, 4 h, and 6 h at room temperature using BacTiter-Glo^®^ Luciferase reagent. (B) The viability of yeast cells upon incubation at pH 5.5 with varied overall concentrations of CHX-loaded shellac NPs at room temperature upon 15 min, 2 h, 4 h, and 6 h of treatment time using Fluorescein Diacetate (FDA) assay. (C) The viability of algal cells upon incubation with varying concentrations of CHX-loaded shellac NPs at room temperature upon 15 min, 2 h, 4 h, and 6 h of incubation time at pH 5.5 using FDA assay, (*n* = 3 for all data points. No ODTAB was used on the shellac NPs, Figure S4: SEM images of *E.coli*. (A) Control sample of *E.coli* and (B) *E.coli* that incubated with 0.005 wt% free CHX-DG after 4 h of treatment, (C,D) *E.coli* incubated for 4 with 0.005 wt% of CHX-loaded shellac NPs. All experiments were conducted at 25 °C. No ODTAB was used to coat on the shellac NPs, Figure S5: SEM images of yeast cells. (A,B) Control sample of yeast cell and (C,D) yeast cells that were incubated with 0.005 wt% free CHX for 4 h, (E,F) yeast cell incubated for 4 h with 0.005 wt% of CHX loaded shellac NPs. No ODTAB was used to coat the shellac NPs, Figure S6: SEM images of *C. reinhardtii* cells. (A,B) A control sample of the *C. reinhardtii* microalgae cells. (C,D) *C. reinhardtii* cells incubated with a solution of 0.005 wt% free CHX for 4 h. (E,F) *C. reinhardtii* microalgae cells incubated with 0.005 wt% CHX-loaded shellac NPs for 4 h. No ODTAB was used on the shellac NPs in this experiment, Figure S7: TEM images of (A) non-loaded shellac NP and (B) CHX-loaded shellac NPs. Samples were prepared and redispersed in deionized water. (A) non-loaded shellac NPs for a solution consisting of 0.25 wt% shellac with 0.2 wt% P407. (B) CHX-loaded shellac NPs consisting of 0.25 wt% shellac with 0.2 wt% P407 and 0.05 wt% CHX dispersed in deionized water, Table S1: The drug loading percent of four different concentrations of CHX-DG.
