# Supplementary material for: Enhanced Antimicrobial Action of Chlorhexidine Loaded in Shellac Nanoparticles with Cationic Surface Functionality
Source: Pharmaceutics. 2021 Sep 2;13(9):1389. doi: 10.3390/pharmaceutics13091389 (PMC8470920; doi:10.3390/pharmaceutics13091389)
Supplement: Supplementary file 1 [file pharmaceutics-13-01389-s001.zip › pharmaceutics-1343599-SI.pdf]

pharmaceutics

Supplementary Material: Enhanced Antimicrobial Action of Chlorhexidine Loaded in Shellac Nanoparticles with Cationic Surface Functionality

Saba S. M. Al-Obaidy, Gillian M. Greenway, and Vesselin N. Paunov

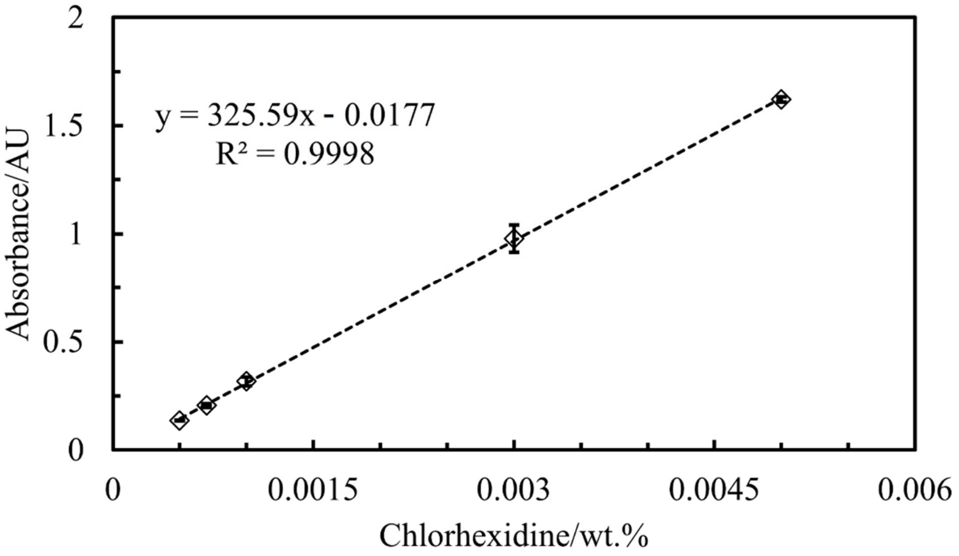

Figure S1. The UV-vis calibration curve at 255 nm of different concentrations of chlorhexidine digluconate (CHX-DG) in deionized water, ($n = 3$).

Citation: Al-Obaidy, S.S.M.; Greenway, G.M.; Paunov, V.N. Enhanced Antimicrobial Action of Chlorhexidine Loaded in Shellac Nanoparticles with Cationic Surface Functionality. *Pharmaceutics* **2021**, *13*, 1389. https://doi.org/10.3390/pharmaceutics13091389

Academic Editor: Ivana Cacciatore

Received: 2 August 2021
Accepted: 29 August 2021
Published: 2 September 2021

Publisher's Note: MDPI stays neutral with regard to jurisdictional claims in published maps and institutional affiliations.

Copyright: © 2021 by the authors. Licensee MDPI, Basel, Switzerland. This article is an open access article distributed under the terms and conditions of the Creative Commons Attribution (CC BY) license (http://creativecommons.org/licenses/by/4.0/).

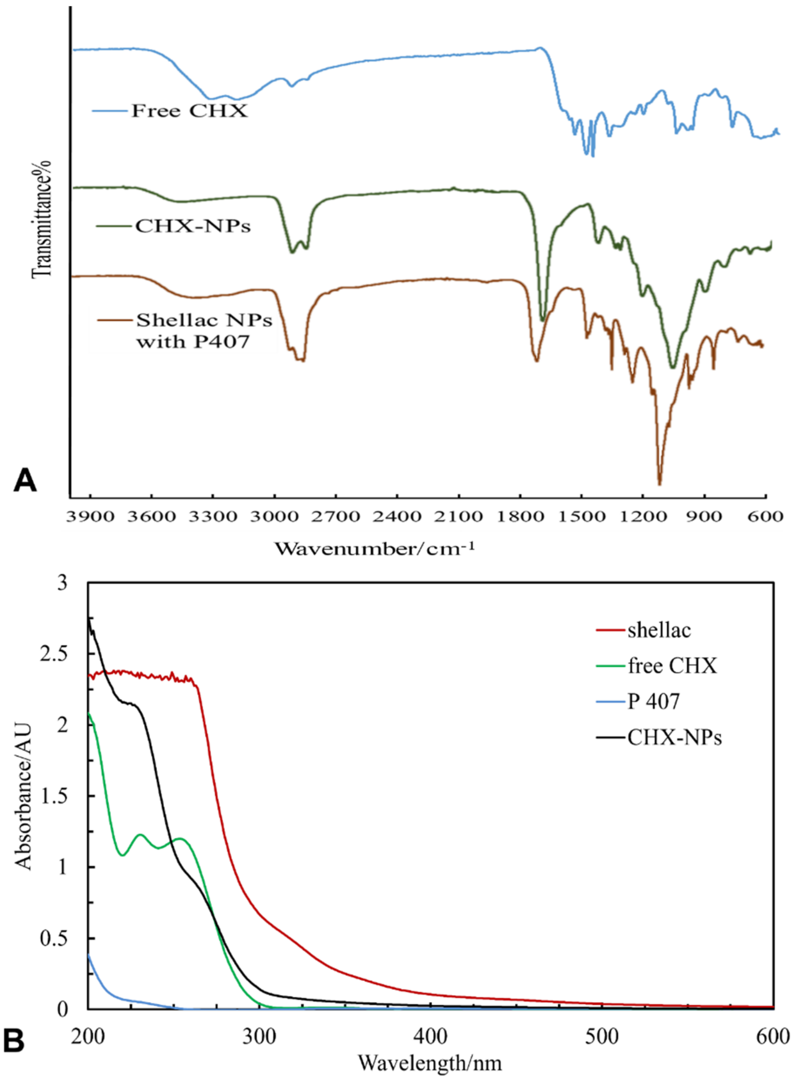

Figure S2. (**A**) The Fourier Transform-IR spectrum of chlorhexidine, 0.03 wt.%. CHX-loaded shellac NPs, and shellac NPs. (**B**) The absorption spectrum of chlorhexidine, free shellac, chlorhexidine loaded shellac NPs and Poloxamer 407 using UV-Vis spectrophotometry.

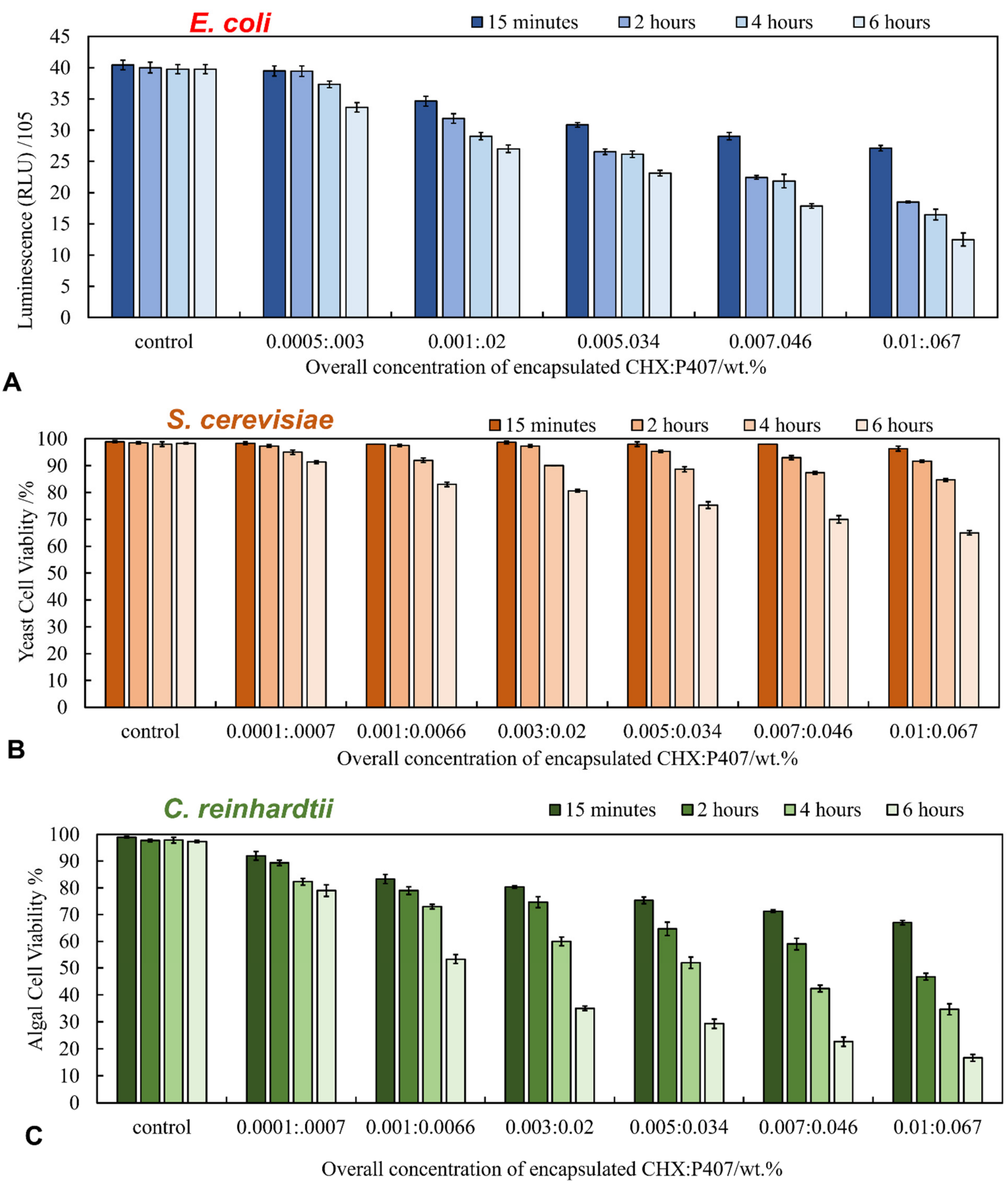

Figure S3. (**A**) The relative luminescence unit representing *E.coli* cells viability incubated at pH 5.5 with different overall concentrations of CHX-loaded shellac NPs at different incubation time 15 min, 2 h, 4 h, and 6 h at room temperature using BacTiter-Glo® Luciferase reagent. (**B**) The viability of yeast cells upon incubation at pH 5.5 with varied overall concentrations of CHX-loaded shellac NPs at room temperature upon 15 min, 2 h, 4 h, and 6 h of treatment time using Fluorescein Diacetate (FDA) assay. (**C**) The viability of algal cells upon incubation with varying concentrations of CHX-loaded shellac NPs at room temperature upon 15 min, 2 h, 4 h, and 6 h of incubation time at pH 5.5 using FDA assay, (n = 3 for all data points. No ODTAB was used on the shellac NPs.

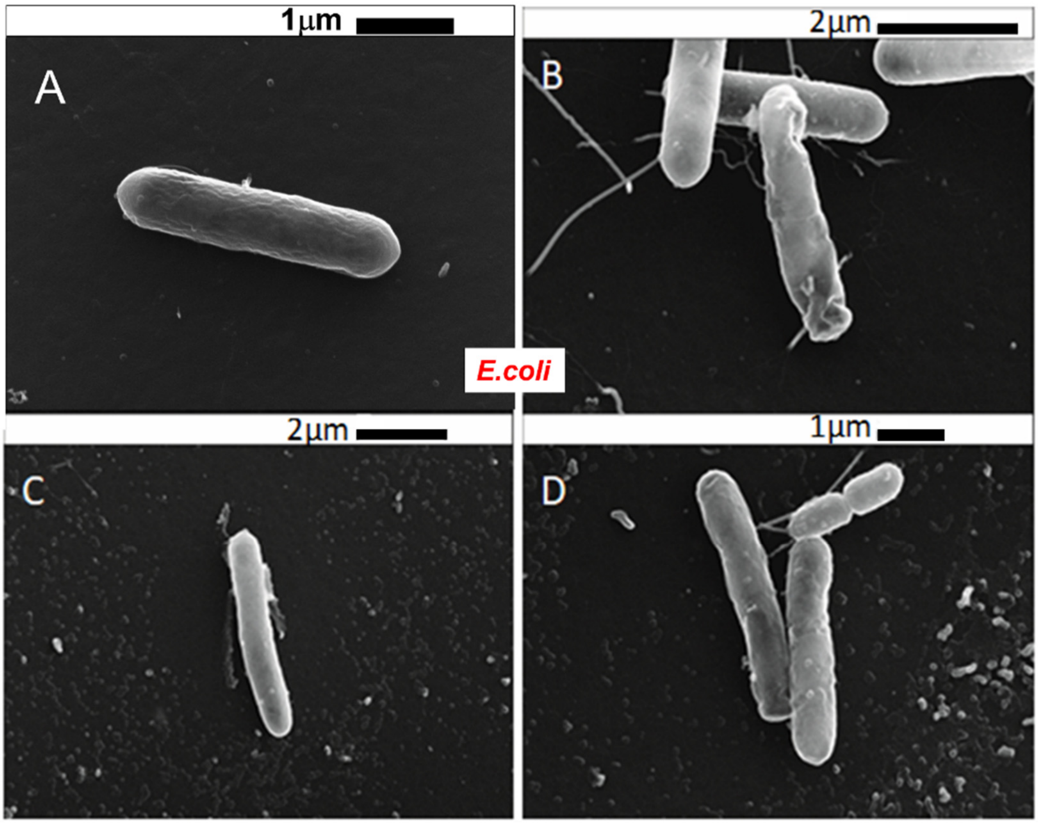

Figure S4. SEM images of *E.coli*. (**A**) Control sample of *E.coli* and (**B**) *E.coli* that incubated with 0.005 wt.% free CHX-DG after 4 h of treatment, (**C,D**) *E.coli* incubated for 4 with 0.005 wt.% of CHX-loaded shellac NPs. All experiments were conducted at 25 °C. No ODTAB was used to coat on the shellac NPs.

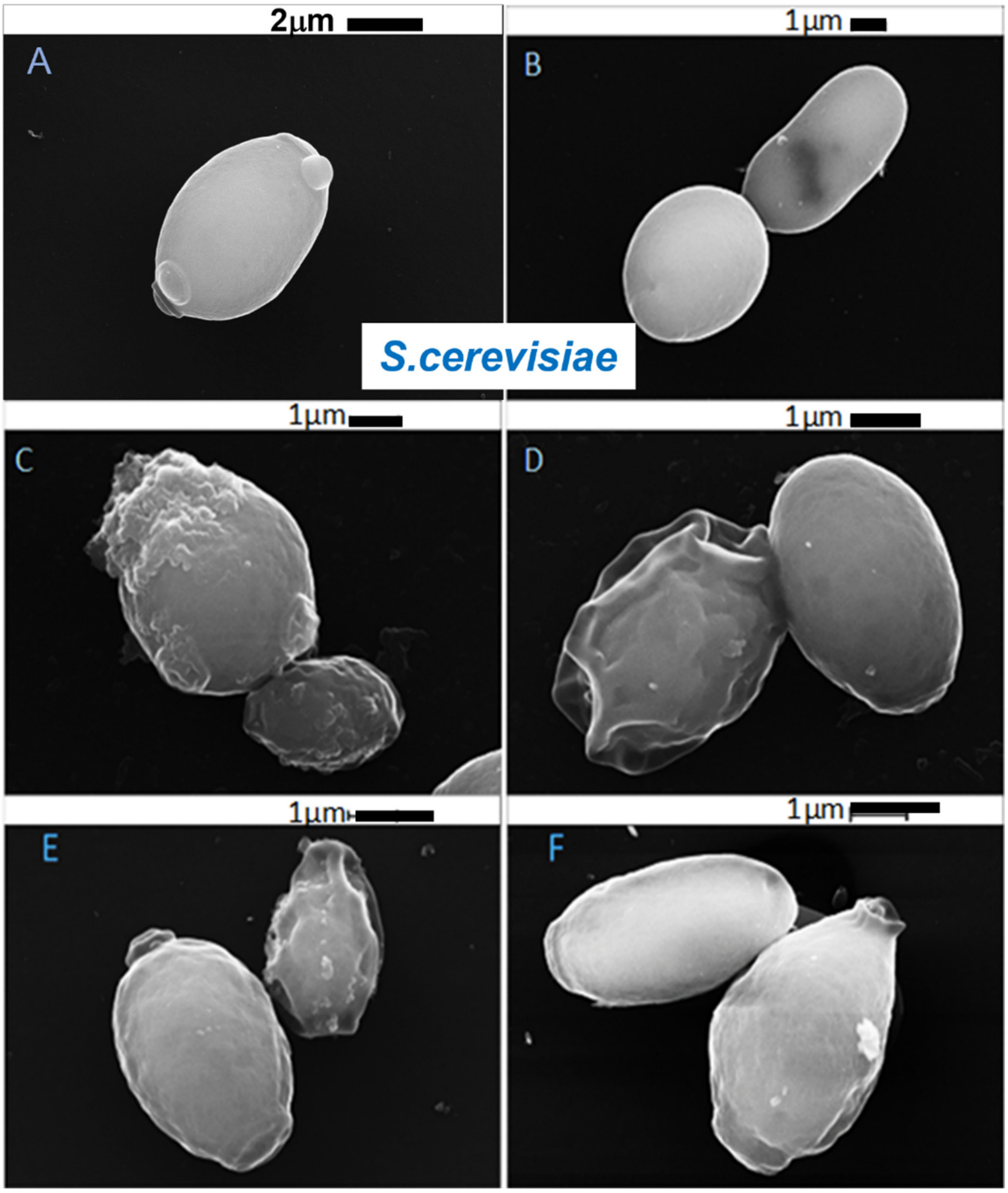

Figure S5. SEM images of yeast cells. (**A**,**B**) Control sample of yeast cell and (**C**,**D**) yeast cells that were incubated with 0.005 wt.% free CHX for 4 h, (**E**,**F**) yeast cell incubated for 4 h with 0.005 wt.% of CHX-loaded shellac NPs. No ODTAB was used to coat the shellac NPs.

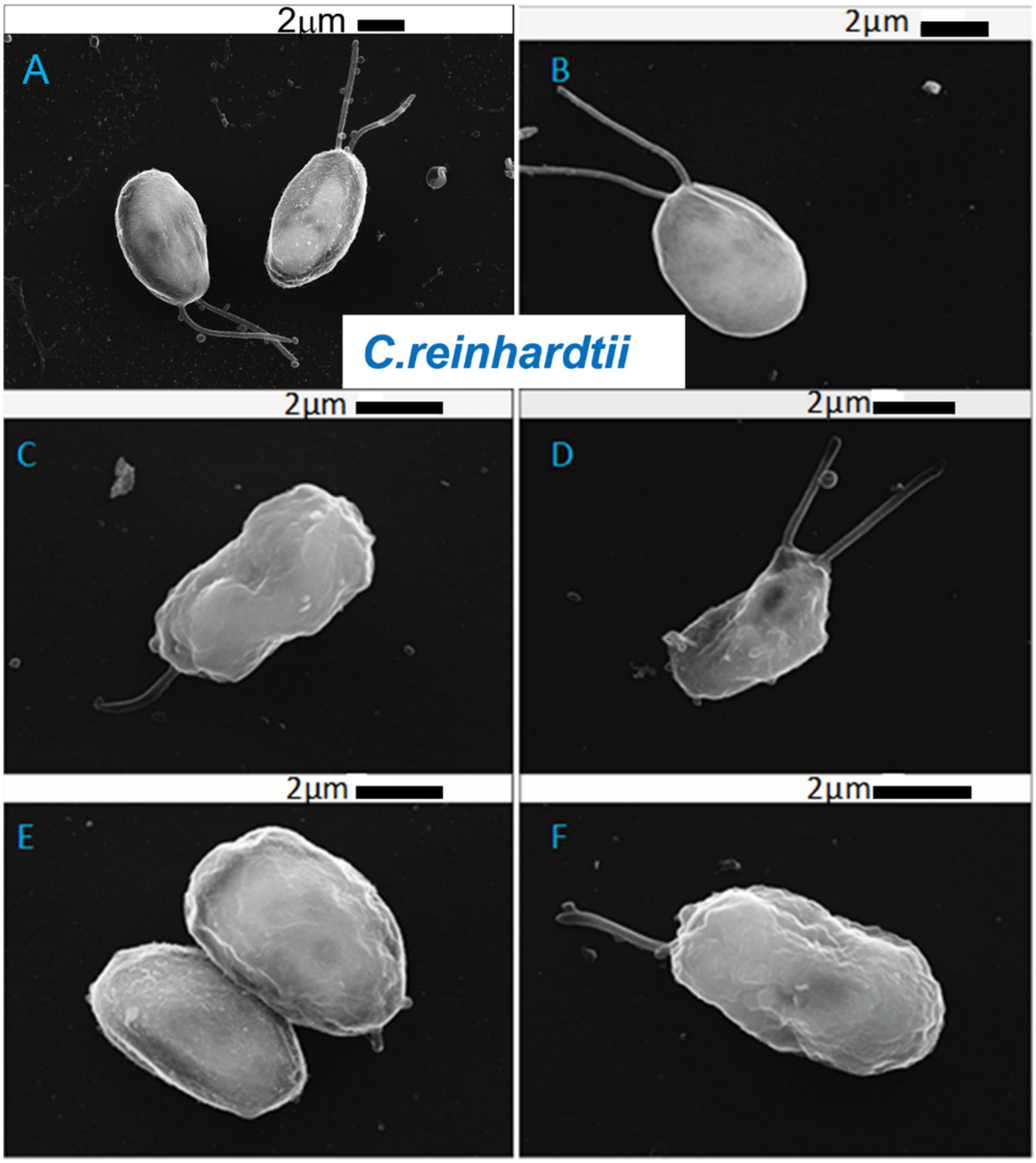

Figure S6. SEM images of *C. reinhardtii* cells. (**A,B**) A control sample of the *C. reinhardtii* microalgae cells. (**C,D**) *C. reinhardtii* cells incubated with a solution of 0.005 wt.% free CHX for 4 h. (**E, F**) *C. reinhardtii* microalgae cells incubated with 0.005 wt.% CHX-loaded shellac NPs for 4 h. No ODTAB was used on the shellac NPs in this experiment.

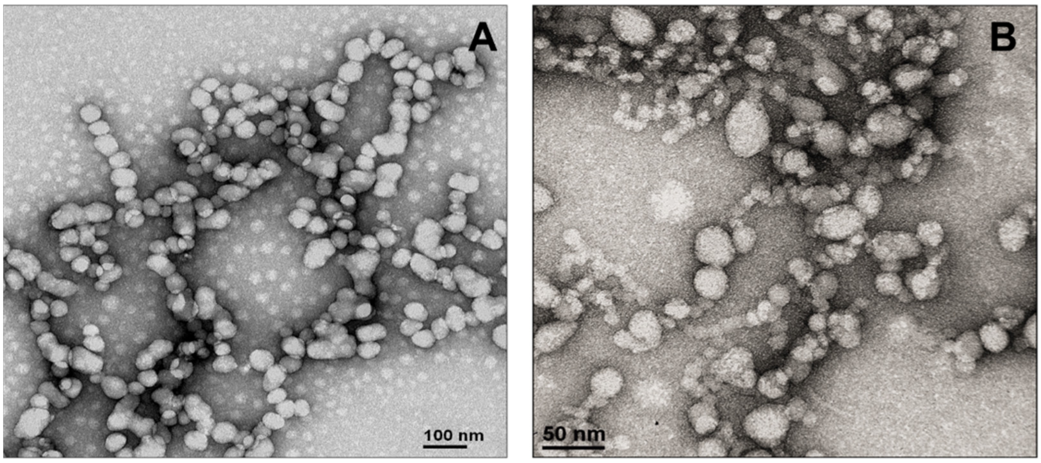

Figure S7. TEM images of (**A**) non-loaded shellac NP and (**B**) CHX-loaded shellac NPs. Samples were prepared and redispersed in deionized water. (**A**) non-loaded shellac NPs for a solution consisting of 0.25 wt. % shellac with 0.2 wt.% P407. (**B**) CHX-loaded shellac NPs consisting of 0.25 wt. % shellac with 0.2 wt.% P407 and 0.05 wt.% CHX dispersed in deionized water.

Table S1. The drug loading percent of four different concentrations of CHX-DG.

CHX Concentration/mol mL^{-1}	Drug Loading (%)
1.11×10^{-7}	1.7
3.3×10^{-7}	9.3
4.46×10^{-7}	12.8
5.57×10^{-7}	16
